# The depletion thickness in solutions of semi-flexible polymers near colloidal surfaces: analytical approximations

**DOI:** 10.1039/d1cp05026e

**Published:** 2022-02-01

**Authors:** C. M. Martens, S. H. M. van Leuken, J. Opdam, M. Vis, R. Tuinier

**Affiliations:** Laboratory of Physical Chemistry, Department of Chemical Engineering and Chemistry, Eindhoven University of Technology P.O. Box 513 5600 MB Eindhoven The Netherlands m.vis@tue.nl r.tuinier@tue.nl; Institute for Complex Molecular Systems, Eindhoven University of Technology P.O. Box 513 5600 MB Eindhoven The Netherlands

## Abstract

We derive a simple, yet accurate approximate mean-field expression for the depletion thickness *δ*_sf_ of a solution of dilute semi-flexible polymers next to a hard surface. In the case of a hard wall this equation has the simple form *δ*_sf_ = *δ*_0_[1 − tanh(*p*_sf_/*δ*_0_)], where *p*_sf_ accounts for the degree of flexibility and *δ*_0_ is the depletion thickness in the case of fully flexible polymers. For fixed polymer coil size, increasing the chain stiffness leads to a decrease in the depletion thickness. The approach is also extended to include higher polymer concentrations in the semidilute regime. The analytical expressions are in quantitative agreement with numerical self-consistent field computations. A remarkable finding is that there is a maximum in the depletion thickness as a function of the chain stiffness in the semidilute concentration regime. This also means that depletion attractions between colloidal particles reach a maximum for a certain chain stiffness, which may have important implications for the phase stability of colloid–polymer mixtures. The derived equations could be useful for the description of interactions in- and phase stability of mixtures of colloids and semi-flexible polymers.

## Abstract 2

The depletion thickness of a solution of semi-flexible polymers reaches a maximum as a function of chain stiffness in the semidilute concentration regime.

## Graphical abstract

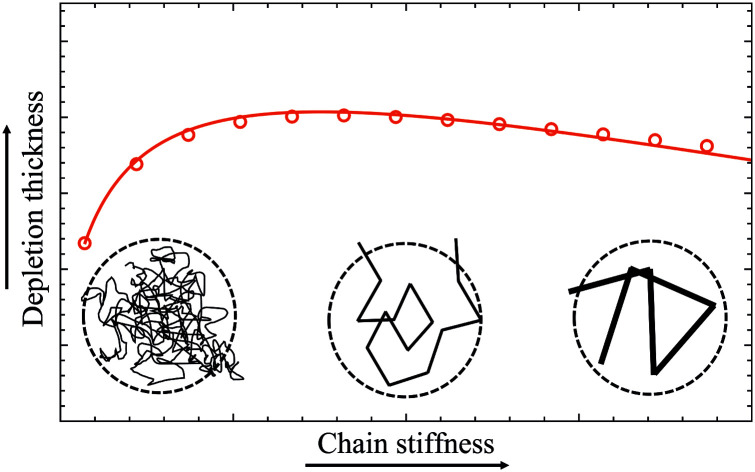

## 1 Introduction

Non-adsorbing polymers induce a net attraction between colloidal particles, often termed the depletion interaction. Upon exceeding a particular polymer concentration, the depletion interaction can lead to phase separation of colloid–polymer mixtures into polymer- and colloid-rich phases.^1,2^ This phase separation is often unwanted in colloid–polymer mixtures, such as paints and food emulsions,^3^ but can be used beneficially in for example the shape and size selection of synthetic colloids^4^ and the fractionation of proteins.^5,6^

The depletion interaction originates from the fact that polymers have less configurational entropy close to a surface,^7^ leading to so-called depletion zones,^8^ which are regions near surfaces that are depleted of polymer. When two depletion zones of adjacent surfaces overlap, an unbalanced osmotic force between the colloidal particles pushes them together,^7–9^ indicating the non-adsorbing polymers induce an effective attraction between the colloidal particles, even though all interactions in the system are purely repulsive.

To quantify the depletion interaction and the resulting phase behavior, the depletion thickness is an important parameter. It quantifies the negative adsorption near a surface and is defined as *δ* = −*Γ*/*φ*_b_, where *Γ* is the adsorbed amount and *φ*_b_ is the bulk polymer concentration. The depletion thickness depends on the polymer segment density profile near the colloidal particles. These polymer segment density profiles may be calculated for instance using computer simulations,^10,11^ numerical computations^12–14^ or analytical approximations.^15–17^ Eisenriegler^15^ showed that for ideal polymers the depletion thickness is close to the polymer radius of gyration *R*_g_: 
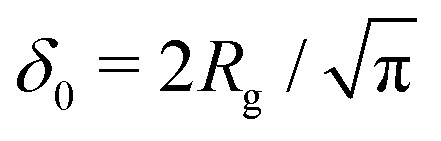
. In contrast, de Gennes^17^ showed that in the semidilute polymer concentration regime, the depletion thickness is only a function of the bulk polymer concentration and the solvent–monomer interactions. Fleer *et al.*^18^ connected these limits and derived an expression that provides a smooth cross-over between the dilute and semidilute concentration regimes.

The results mentioned hold for flexible polymers only, in which the monomer size *l* is equal to the Kuhn length *b*. In practice, though, most polymers exhibit some form of intrinsic chain stiffness, which increases the effective Kuhn length of the polymer segments and in turn enlarges the radius of gyration of the polymer.^19,20^ Additionally, because of the intrinsic stiffness, semi-flexible polymers have less configurational entropy compared to fully flexible polymers.^21^ This may suggest that due to the entropic nature of depletion, chain stiffness possibly affects the polymer segment density profiles and the resulting thickness of the depletion layer. This hypothesis was previously confirmed by Yamazaki *et al.*^22^ in an experimental system comprising fumed silica particles and either flexible or semi-flexible polymers. They found that the phase stability strongly depends on the chain flexibility. A notable effect was that a higher polymer concentration is required to induce phase separation in the case of polymers with a larger chain stiffness, *i.e.*, stiffer chains with a comparable radius of gyration are less effective depletants. They argued that this flexibility dependence is attributed to the entropy difference between a flexible and semi-flexible polymer, causing a decrease in the strength of the depletion interaction. Numerous other studies confirmed the chain stiffness dependence of the depletant efficiency.^23–28^

This raises the question: what is the effect of chain stiffness on the depletion thickness of polymer solutions? Ausserré *et al.*^29^ used geometrical arguments to derive that the depletion thickness of a solution of semi-flexible polymers *δ*_sf_ has the following form: *δ*_sf_ = *δ*_0_ − *b*, where *b* is the effective (Kuhn) segment length. The depletion thickness of a solution of semi-flexible polymers is thus smaller than a solution of flexible polymers by an amount of the order of the Kuhn length *b*. Later work by Lue,^30^ who derived a Padé approximation between the excluded volume of a flexible polymer and a stiff needle around a sphere, showed that the excluded volume between a polymer and a sphere is smaller for stiffer polymers if the radius of gyration is kept constant.

The study presented here is motivated by the following observations: both the theoretical treatment of Ausserré *et al.*^29^ and Lue^30^ are derived for dilute polymer solutions only, while the concentration effects in the semidilute regime play an important role in the depletion thickness.^18^ Additionally, as shown in Appendix A, even though these theoretical approaches are qualitatively insightful, they are quantitatively inconsistent with numerical self-consistent field (SCF) computations. Furthermore, although the density profiles and depletion thickness for semi-flexible polymers can be computed using numerical self-consistent field theory,^13,31,32^ analytical expressions are useful to estimate other physical properties such as the surface tension^17,33^ and phase behavior of colloid–polymer mixtures.^2,8^ Additionally, it may for instance, yield analytical expressions of the depletion attraction between both flat walls and spheres,^8^ and the friction coefficient of colloidal particles diffusing through a semi-flexible polymer solution.^34,35^

Hence in this paper, we aim at finding generalized analytical expressions that enable the quantification of the effect of chain stiffness on the depletion thickness of polymer solutions in both dilute and semidilute concentrations near a flat wall and a spherical colloidal surface. The central concept of the derivation is based on the mapping of known continuum expressions on a lattice theory for semi-flexible polymers. The analytical results obtained are compared to numerical SCF computations, which are performed using the Scheutjens–Fleer formalism.^12,13^ All results shown are for a *θ*-solvent, but they can be generalized to a good solvent using straightforward extensions^18,36^ as demonstrated in Appendix C. Furthermore, all length-scales are given in units of the the bond length *l*.

The outline of this manuscript is as follows. In Section 2, we explain the lattice-based self-consistent field theory for semi-flexible chains, which is used as a starting point for our derivation but is also used to compare our results. We introduce the propagators for a lattice chain with variable stiffness, which are later used to derive boundary conditions for the analytical approximation. In Section 3 analytical expressions are derived using the lattice model equations and a continuum theory for the polymer segment density profiles. In Section 4, the results of our approximate theory are compared with numerical self-consistent field computations and discussed. Finally, the main conclusions are summarized in Section 5.

## 2 Theoretical background

### 2.1 Self-consistent field theory

The theoretical treatment presented here was first described by Leermakers and colleagues^31^ and later expanded upon by Wijmans *et al.*^32^ It must be noted that it is a mean-field theory, which implies that the polymer chains in the bulk behave as ideal Gaussian chains. For a *θ*-solvent this is correct, however, for good-solvent conditions fluctuations are not accurately accounted for. The scaling exponents obtained from SF-SCF thus differ from refined field-theoretical methods such as renormalization group theory.^18,37^

We focus on a mixture composed of semi-flexible homopolymers and solvent molecules near a hard wall. Concentration gradients are accounted for in a single direction only. The lattice consists of *M* layers of thickness equal to the monomer length *l*. Let *z* denote the layers as *z* = 1, 2, 3,…, *M*. The solvent molecules have the same size *l* and volume *l*^3^ as the monomers. A surface is placed in the boundary layer next to the first layer at *z* = 0. Let *λ*_0_ be the fraction of nearest neighbor contacts in the same layer and *λ*_1_ be the fraction of nearest neighbor contacts in the next or previous layer, so *λ*_0_ + 2*λ*_1_ = 1. The polymer statistics as shown here are for a flat geometry, but extensions towards spherical geometry are straightforward.^13^

#### 2.1.1 Potential field

A lattice polymer chain is composed of *N* segments, each with ranking number *s* = 1, 2, 3,…, *N*. The statistical weighting factor of segment *s* in layer *z* is simply the Boltzmann weight due to the potential experienced by the segment in that layer:

1 *G*(*z*) = e^−*u*_*z*_^,

where *u*_*z*_ is the potential in layer *z* in units of *k*_B_*T*. For a polymer in a monomeric solvent this potential follows from Flory–Huggins theory as:^1,12,13^2
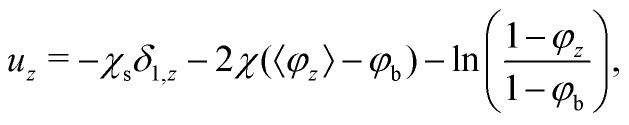
where *χ*_s_ is the adsorption energy, supplemented with a Kronecker delta to ensure that only in the first layer the adsorption energy is applied, *χ* is the Flory–Huggins interaction parameter between the solvent and segment, and *φ*_b_ is the volume fraction of polymer segments in the bulk solution. We follow the convention of Fleer *et al.*^38^ and define the adsorption energy to be positive for repulsion and negative for attraction. The quantity 〈*φ*_*z*_〉 is defined as the contact fraction:^1,12^ 〈*φ*_*z*_〉 = *λ*_1_*φ*_*z*−1_ + *λ*_0_*φ*_*z*_ + *λ*_1_*φ*_*z*+1_. The potential *u*_*z*_ has a direct effect on the statistics of the polymer chain, which will be treated in the next section.

#### 2.1.2 Polymer statistics

Consider segment *s* to be positioned on a cubic lattice and connected to segment *s* − 1. Let *j* = −1, 0, 1 identify the direction on the lattice: *j* = −1 for a direction on the lattice from *z* to *z* − 1, *j* = 0 for the four directions within the same layer *z* and *j* = 1 for the direction from *z* to *z* + 1. Three coupled step-weighting probabilities, *P*_f_, *P*_b_ and *P*_p_, corresponding to a forward, backwards and perpendicular step, are defined for each direction. These weighting probabilities are coupled as *P*_b_ + 4*P*_p_ + *P*_f_ = 1. For lattice polymer chains with finite stiffness the backfolding weighting probability *P*_b_ is zero.^32^ Thus, all conformations of the polymer chain where backfolding occurs are excluded. Wijmans *et al.*^32^ derived that for a cubic lattice, the Kuhn length *b* of a polymer composed of segments with length *l* is related to *P*_f_ through:3
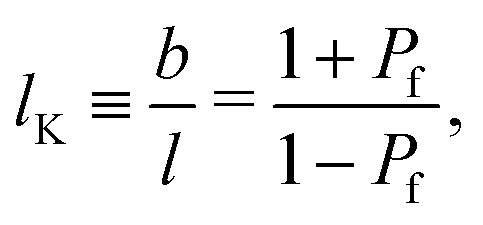
where *l*_K_ is the (normalized) Kuhn length of the polymer. Eqn (3) has the limiting values of *l*_K_ → ∞ for *P*_f_ = 1 and *l*_K_ = 1 for *P*_f_ = 0, hence, finite semi-flexibility can be mimicked for 0 < *P*_f_ < 1.

To include the directionality into a recurrence relation, the statistical weights *G*(*z*,*s*,*j*|1) and *G*(*z*,*s*,*j*|*N*) are introduced. In *G*(*z*,*s*,*j*|1) the variable *j* is the direction from segment *s* to *s* + 1, thus *G*(*z*,*s*,*j*|1) is the probability to find segment *s* in layer *z* that will make a step in the direction *j*. In contrast, in *G*(*z*,*s*,*j*|*N*), *j* is the direction from segment *s* + 1 to segment *s*. Consequently, *G*(*z*,*s*,*j*|*N*) is the probability to find segment *s* in layer *z* coming from the direction *j*. On a cubic lattice, the end-segment probability is the average over all six directions *j*:^13^4a
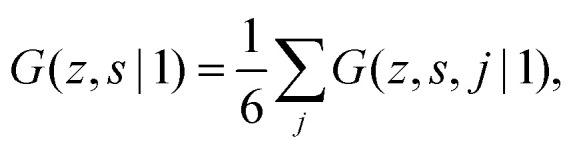
4b
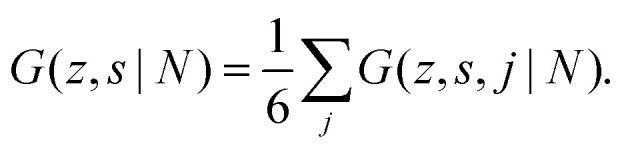
The propagator for a segment *s* that will make a step in the direction of *j* is given by a second-order Markov approximation:^32,39^5a
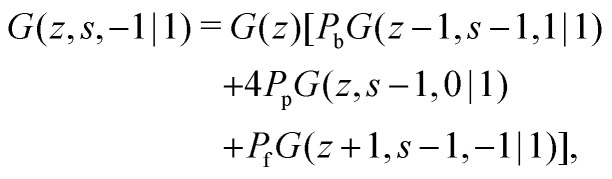
5b
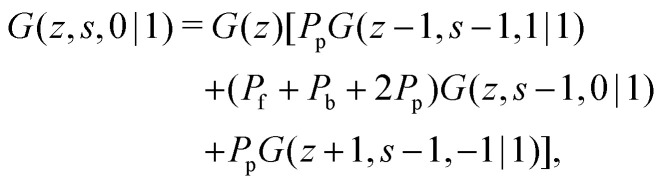
5c
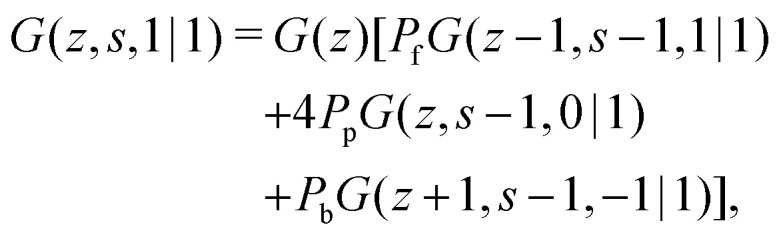
with starting condition *G*(*z*,1,*j*|1) = *G*(*z*). If one starts from the other side of the chain, it is important to realise that now *j* is defined as the direction from *s* + 1 to *s*. This creates an asymmetry in the propagators because in *G*(*z*,*s*,*j*|1) the directionality is between segment *s* − 1, *s* and *s* + 1 (*j* is the direction from *s* to *s* + 1) and *G*(*z*,*s*,*j*|*N*) the directionality is between segment *s*, *s* + 1 and *s* + 2 (*j* is from *s* + 1 to *s*). Starting with *G*(*z*,1,*j*|*N*) = *G*(*z*), the propagator for *G*(*z*,*s*,*j*|*N*) becomes:^32,39^6a
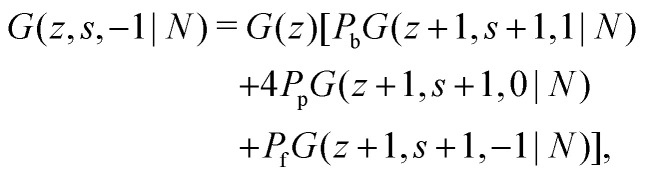
6b
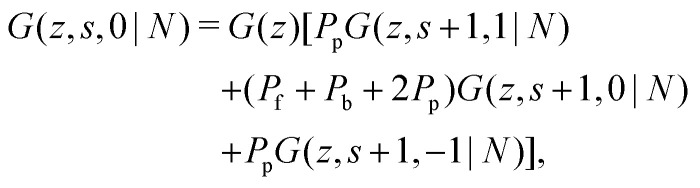
6c
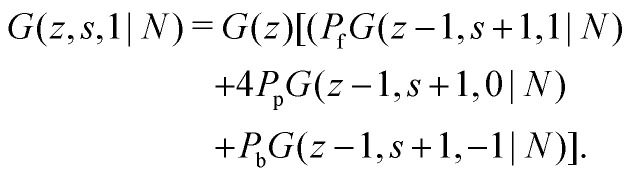
The volume fraction profiles of segment *s* and the polymers can be found using the composition law:^13,31,32^7
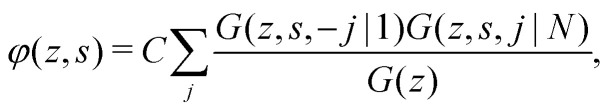
where *C* is a normalization constant equal to *φ*_b_/6*N*. Summing over all segments *s* the local polymer segment volume fraction is obtained at layer *z*. The self-consistency of the theory now also becomes clear; the segment potentials depend on the volume fractions, and the volume fractions also depend on the segment potentials, thus creating a set of equations that should be solved in a self-consistent way.

### 2.2 Self-consistent field computations

The equations presented in the previous section are solved numerically using the sfbox software package. As initial conditions, the polymer bulk volume fraction, *i.e.*, the volume fraction located somewhere in the bulk, outside of the lattice, is chosen. Additionally, the monomer-solvent interaction parameter *χ*, the adsorption energy *χ*_s_, the number of monomers *N*, and the forward probability *P*_f_ are set. In this paper, we only considered a *θ*-solvent, for which *χ* = 0.5 and used a cubic lattice for both a flat and spherical geometry, where *λ*_1_ = 1/6. The free energy of the system is then minimized using an iterative scheme, yielding the partition function of the polymers and subsequently giving the equilibrium volume fractions at each lattice layer *z*. The SF-SCF calculations slightly depend on the lattice chosen^40^ and one may choose to use a different lattice parameter. However, the equations presented must then be adjusted, as the transition probabilities and the relation between the Kuhn length and the forward probabilities are dependent on the lattice parameter.

## 3 Analytical approximation for semi-flexible polymers

In this section we derive analytical approximations for the segment density profile and depletion thickness of a solution of semi-flexible polymers near a non-adsorbing surface. This is done by combining the lattice theory presented in the previous section with a modified analytical expression for the segment density profile of solutions of flexible polymers.

Fleer *et al.*^18^ showed that the normalized polymer segment density *ρ* = *φ*/*φ*_b_ of a dilute solution of depleted flexible polymers can be described accurately by *ρ*(*z*) = tanh^2^(*z*/*δ*_0_), where 
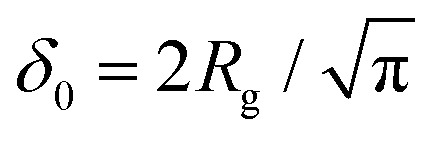
 is the depletion thickness and *z* is now a continuous variable describing the distance from the surface in units of the bond length *l*. Using the argument that more stiff polymers have less configurational entropy to start with,^21,41^ the relative entropy loss due to the non-adsorbing surface is smaller for stiffer polymers. We thus expect that the inhomogeneous part of the density profiles shifts closer to the surface upon increasing the chain stiffness. To account for this, we hypothesize that the segment density has the approximate form: *ρ*(*z*) = tanh^2^[(*z* + *p*)/*δ*_0_], such that the density profile is effectively shifted towards the surface over a length *p* (where *p* is units of the bond-length *l*). This is similar to the form that was proposed by Ausserré *et al.*,^29^ where they used *p* = *l*_K_. In the following section, we derive an analytical expression for *p* by mapping the proposed continuum expression on the lattice theory through boundary conditions at the surface.

### 3.1 Lattice boundary condition for semi-flexible chains

Consider a polymer segment in the layer next to the surface. After letting the probabilities of terms which contain a direction from or towards the surface go to 0, and excluding all terms in which a segment is contained within the wall (*z* = 0), we obtain from eqn (4) and (5):8
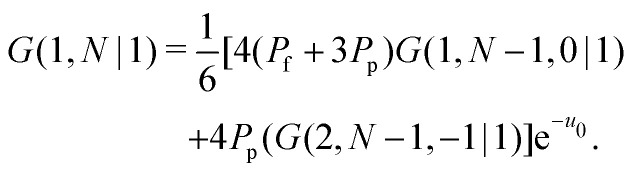
As noted by Leermakers *et al.*^31^ and Fleer *et al.*,^13^ in SCF a system of sufficiently long semi-flexible chains with *N* monomers of size *l* can be approximated by a system of re-scaled flexible chains with *N*_K_ monomers of size *b*. This approximation implies that the statistical weights in eqn (8) can be approximated by the statistical weights of a re-scaled flexible chain, where the lattice spacing is now equal to the Kuhn length *b*. In normalized units *l*_K_ = *b*/*l* this yields:9
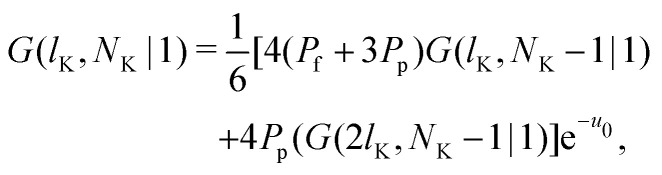
where *G*(*l*_K_,*N*_K_|1) is the propagator for a polymer segment at a normalized distance *l*_K_ from the surface. In principle this approximation is not accurate, as it does not hold in the region with a large concentration gradient, such as close to the surface.^39^ However, we can assume that it is sufficiently accurate, as it allows us to obtain a continuum expression for the boundary conditions, which can be solved analytically. Subsequently, we propose a modified analytical expression for *G*, which yields an analytical expression for the statistical weights and therefore concentration profile of a solution of semi-flexible polymers at a hard wall.

Some further simplifications are needed to obtain a continuum expression. Sufficiently long chains are assumed such that *N*_K_ ≈ *N*_K_ − 1; together with 4*P*_p_ + *P*_f_ = 1 and eqn (3) we obtain the boundary condition:10
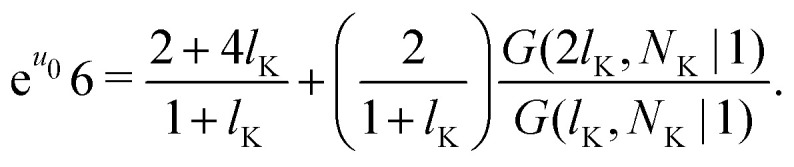
The lattice boundary condition can be translated to a continuum expression as:^38,42^11
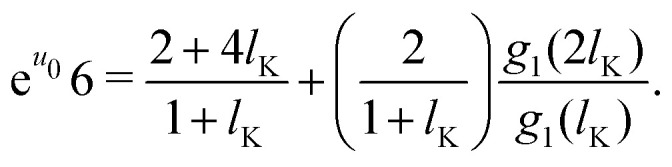
Similarly from the propagator for *G*(1,*s*|*N*) we obtain:12
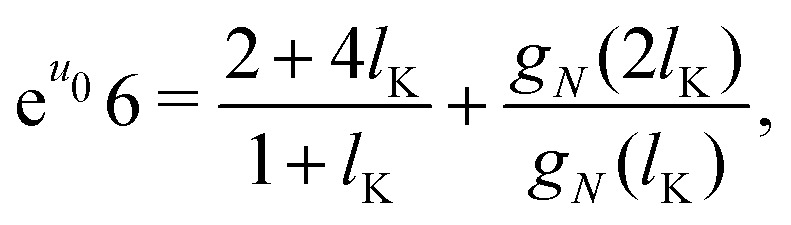
where *g*_*i*_ is the continuum expression for the statistical weight of a polymer with *N*_K_ segments with size *l*_K_, and the subscripts 1 and *N* denote that these functions correspond to a solution with either one of the boundary conditions. In a ground-state approximating manner^13,18,38,43^ we use *g*_*i*_(*z*) ≈ tanh[(*z* + *p*)/*δ*_0_].

The solutions which satisfy these boundary conditions, *g*_1_ and *g*_*N*_, are coupled. In a ground-state approximation the composition law is given by *ρ*(*z*) = *g*(*z*)^2^; combining this with the lattice composition law for semi-flexible chains, eqn (7), we find that we can approximate *g*(*z*)^2^ as *g*_*N*_(*z*)*g*_1_(*z*). Thus the ground-state composition law for a solution of semi-flexible chains then becomes:

13 *ρ*(*z*) = *g*(*z*)^2^ ≈ *g*_*N*_(*z*)*g*_1_(*z*),

which couples eqn (11) and (12). The functions *g*(*l*_K_) and *g*(2*l*_K_) require special consideration. When a continuum model is mapped on a discretized space, the correct values for *z* = *l*_K_ and *z* = 2*l*_K_ are found when shifted half a bond length: *z* = *l*_K_/2 and *z* = 3*l*_K_/2.^18,38,42^ When mapping the continuous functions within the lattice boundary conditions, these values for *z* are used.

### 3.2 Concentration profile at a flat wall for dilute conditions

An explicit expression for the density shift length *p* can be obtained by solving the boundary conditions derived in the previous section. The central assumption in this section is that entropic effects dominate the shape of the depletion concentration profile of semi-flexible polymers. These entropic effects are entirely described by the chain statistics as obtained from the boundary conditions, equations eqn (11) and (12). This assumption allows us to neglect the potential *u* from the boundary conditions and obtain a density shift length that accounts only for the stiffness of the polymer. In other words, we assume that the density shift length derived from the boundary conditions for *u* = 0 holds for all *u* > 0.

Starting with *g*_*i*_(*z*) = tanh[(*z* + *p*_*i*_)/*δ*_0_] and introducing the parameters *p*_1_ and *p*_*N*_, corresponding to the density shift lengths obtained from either one of the boundary conditions, and using *u* = 0 in equations eqn (11) and (12) yields:14a
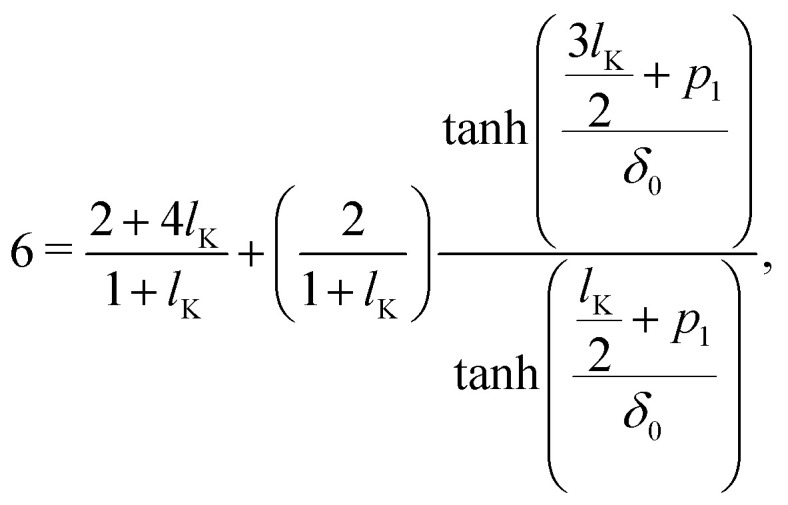
14b
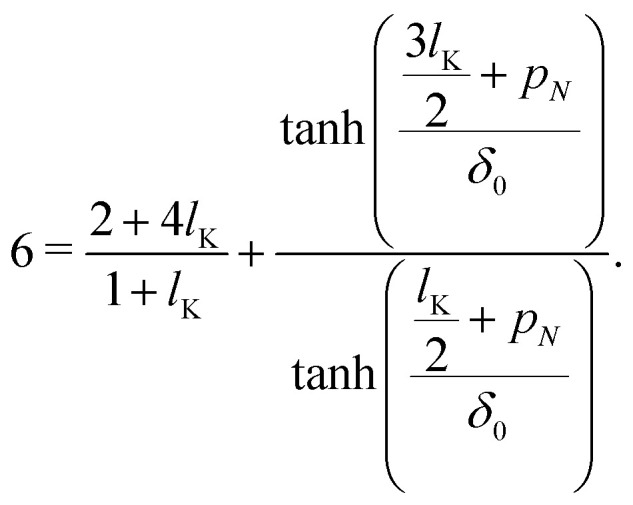
Assuming *p* ≪ *δ*_0_, such that we may approximate tanh(*x*) ≈ *x* and solving for *p*_1_ and *p*_*N*_ gives:15a
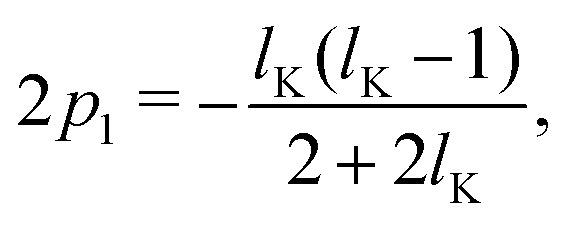
15b
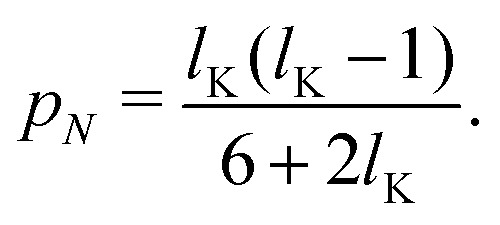
As a final step, *p*_1_ and *p*_*N*_ are connected through the adjusted composition law at *z* = 0 as given in eqn (13). Again assuming *p*_1_,*p*_*N*_,*p* ≪ *δ*_0_ and approximating tanh(*x*) ≈ *x* provides:

16 *p*^2^ ≈ *p*_1_*p*_*N*_.

**Fig. 1:**
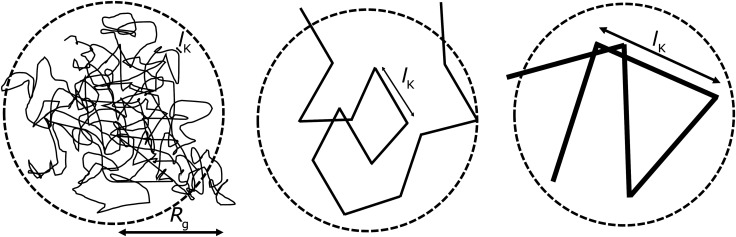
The two relevant length scales for semi-flexible polymers; the radius of gyration *R*_g_ and the Kuhn length *l*_K_ (in units of the monomer length *l*). The radius of gyration is kept constant while the Kuhn length increases from left to right.

Because *p*_1_ < 0 and *p*_*N*_ > 0 for all *l*_K_, *p* is complex valued. To account for this we define *p*_sf_ = |*p*|, where *p*_sf_ denotes the overall density shift length. Using eqn (15a) and (15b) gives the final (real-valued) expression for *p*_sf_ (in units of the bond length *l*):17
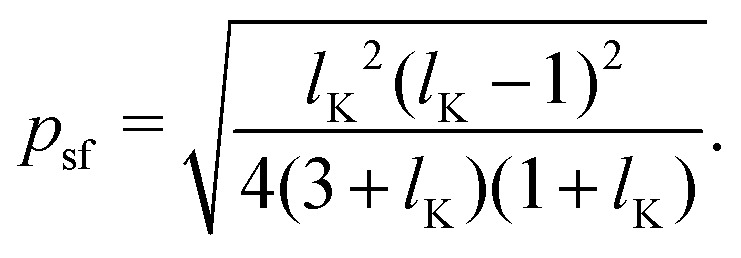
The polymer segment density profile for a solution of semi-flexible chains then becomes:18
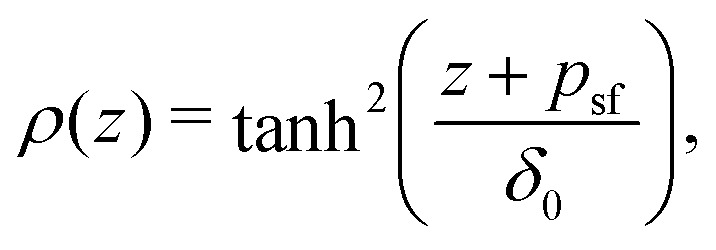
where 
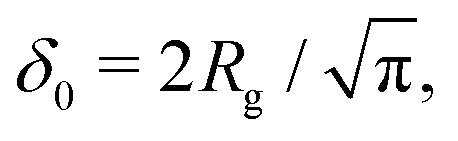
 with *R*_g_ the radius of gyration (in units of the bond length *l*) of a re-scaled Kuhn polymer 
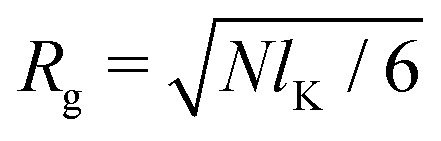
. The length scales *R*_g_ and *l*_K_ = *b*/*l* are schematically shown in Fig. 1; here the chain stiffness is varied at a constant *R*_g_; which is achieved by decreasing the number of monomers.

Because *p*_sf_ is a positive quantity the concentration profile is shifted closer to the surface, as *p*_sf_ increases with increasing chain stiffness. Effectively, this results in a smaller depletion layer for stiffer chains. An analysis of the limiting behavior of eqn (17) reveals that it converges towards *p*_sf_ = *l*_K_/2–3/2 for large Kuhn lengths and *p*_sf_ = 0 for *l*_K_ = 1. Thus, the theory is consistent with the theory for flexible polymers;^16,18^ in the limit of *l*_K_ = 1, *ρ*(*z*) = tanh^2^(*z*/*δ*_0_) is recovered.

### 3.3 Depletion thickness at a flat wall in dilute conditions

From the continuum concentration profile obtained with eqn (18), an expression for the depletion thickness of a solution containing semi-flexible polymers at a flat wall can be derived. The depletion thickness is given by:^18^19
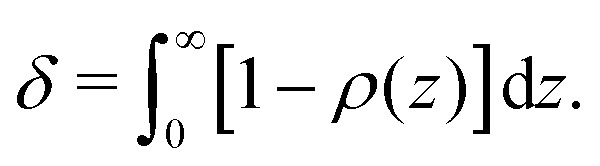
Insertion of eqn (18) for *ρ*(*z*) and carrying out the integration of eqn (19) yields the following expression for the depletion thickness of a solution of semi-flexible chains:20
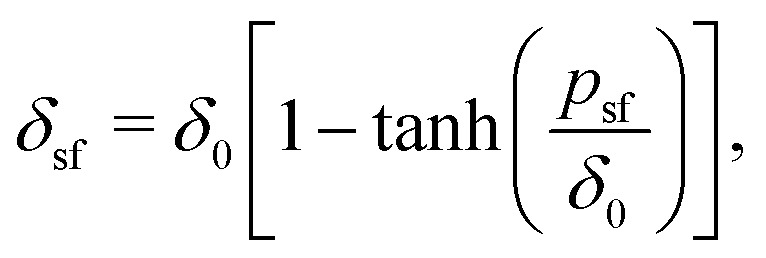
which is valid for dilute polymer solutions.

### 3.4 Generalized depletion thickness

The depletion thickness as shown in eqn (20) is only valid for dilute conditions. Fleer *et al.*^18,44^ derived a generalized (mean-field) expression for the depletion thickness *δ*_g_ which applies to the entire concentration range, including semidilute polymer solutions:21
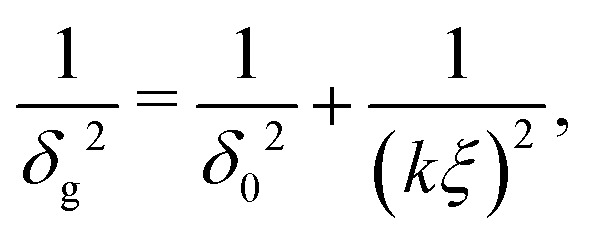
with22a
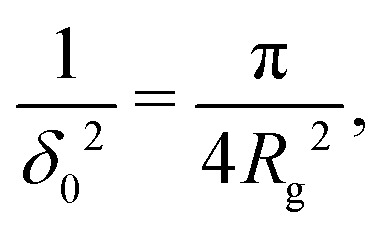
22b
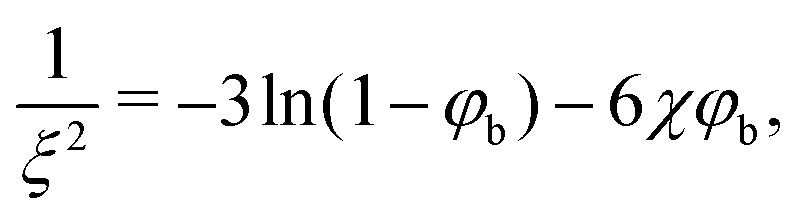
where *ξ* is the (bulk) correlation length in units of the bond length *l*, and *k* is a numerical constant:23
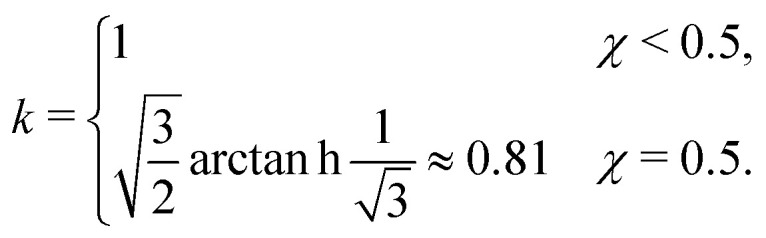


In order to obtain an expression for the correlation length for a solution of semi-flexible chains we apply a Kuhn model approach. The correlation length can be regarded as the size of a blob.^17,18^ Let *N*_B_ be the number of segments inside this blob, the (mean-field) size of this blob is then 
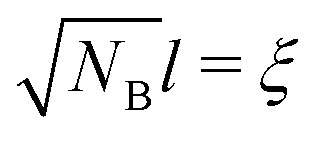
. If we introduce chain stiffness and apply the Kuhn model, we can calculate the number of Kuhn segments in this blob: *N*_B,K_ = *N*_B_/*l*_K_. The size of the blob then becomes 
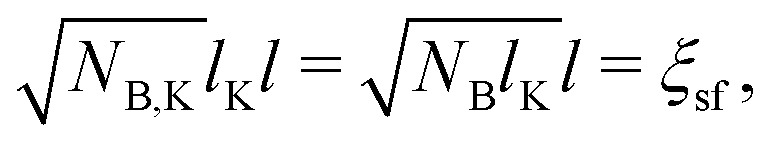
 with *ξ*_sf_ the re-scaled blob size. We can write this as:24
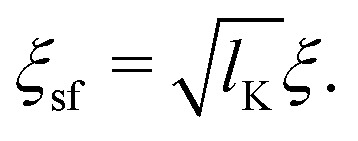
This expression for the bulk correlation length of a semi-flexible polymer solution *ξ*_sf_ is similar to the bulk correlation lengths as derived by Shimada *et al.*^45^ and Marques *et al.*^46^ using random phase approximation theory. These authors found the same (*l*_K_)^1/2^ dependence of the bulk correlation length.

Rewriting eqn (21) and applying eqn (20) yields an expression for the depletion thickness of a solution of semi-flexible chains:25
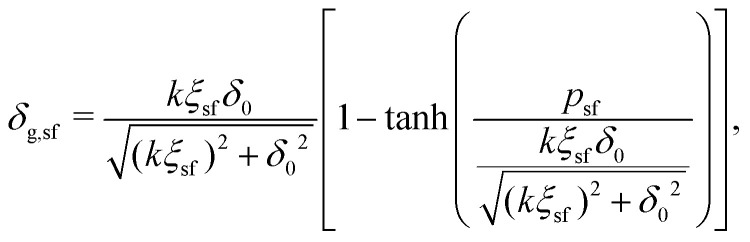
which predicts the depletion thickness over the complete range of polymer segment volume fractions.

### 3.5 Extension to spheres

Eqn (18) describes the density profile of a solution of semi-flexible polymers next to a non-adsorbing wall. An extension towards the depletion profile around a sphere can be made using a similar approach. Two research groups independently found the concentration profile of an ideal, flexible chain near a sphere with radius *a*, where *a* is in units of the bond length *l*:^16,47^26
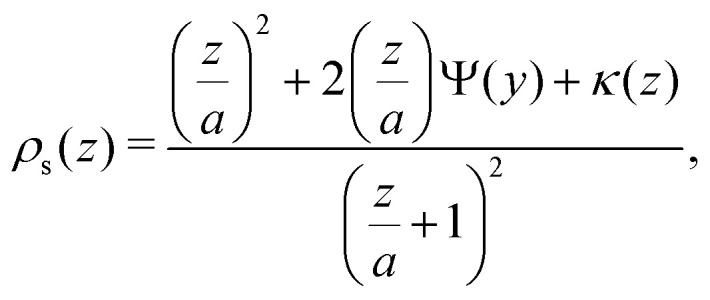
where *κ*(*z*) is defined as

27 *κ*(*z*) = 2*Ψ*(*y*) − *Ψ*(2*y*),

with *y* = *z*/*R*_g_ and *Ψ*(*y*) is given by28
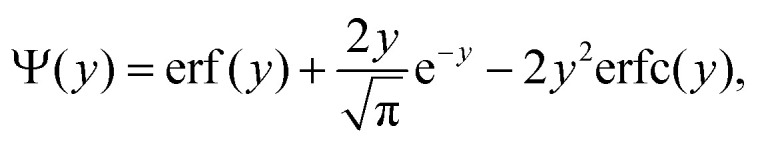
where *z* is now the distance from the surface of the sphere. Fleer *et al.*^18^ showed eqn (26) can be accurately approximated with the following expression:29
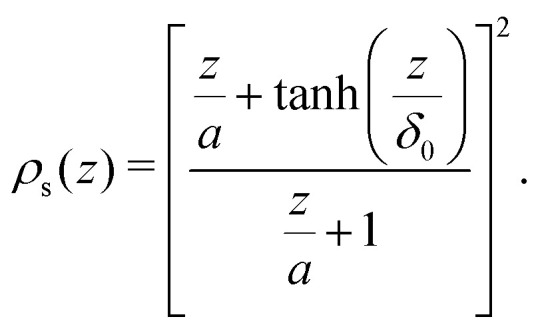
In the limit of a flat plate (*a* → ∞) eqn (29) reduces to *ρ*(*z*) = tanh^2^(*z*/*δ*_0_). Using the Ansatz that, as for the ideal case, the density profile shifts by a length *p*_sf_, we propose the density profile of a solution of semi-flexible polymers around a non-adsorbing sphere of radius *a* is given by:30
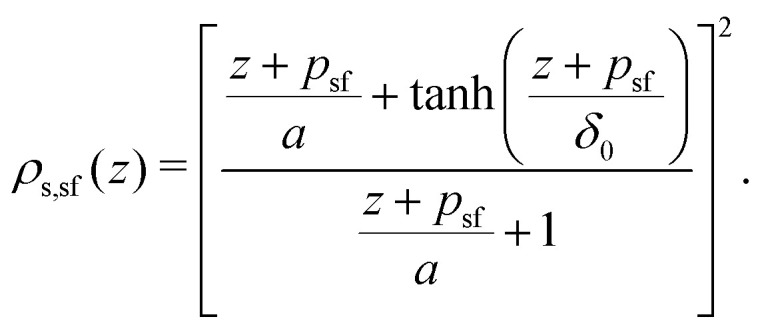


#### 3.5.1 Depletion thickness around a sphere

Using the segment density profile around a sphere we can obtain an expression for the depletion thickness around a sphere. Similarly to the depletion thickness at a flat wall, in a spherical geometry, the negative adsorption again gives the depletion thickness. The depletion thickness for a solution of semi-flexible polymers around a sphere (*δ*_s,sf_) then follows from:^8,18^31
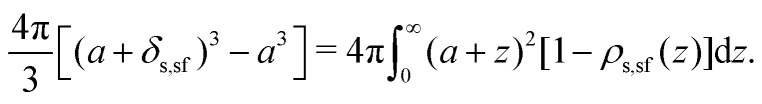
No closed-form solution that we know of exists for this integral, but it can be approximated as elaborated in Appendix B. The depletion thickness of a solution of semi-flexible polymers near a colloidal sphere of radius *a* then becomes:32
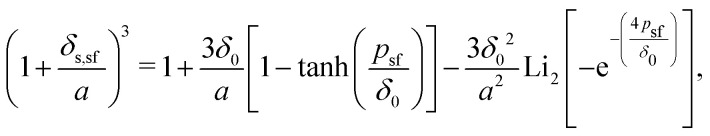
where Li_2_ is the dilogarithm defined as 
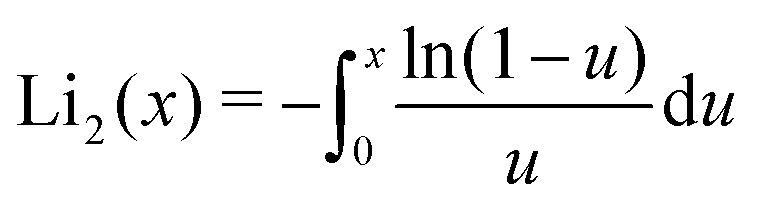
. The depletion thickness around a sphere has the limiting value of 1 + *δ*_0_^2^π^2^/(4*a*^2^) + 3*δ*_0_/*a* for *l*_K_ = 1, which is the same result as obtained by Fleer *et al.*^18^ for a solution of flexible chains. In their paper it was noted that this result is approximate. Aarts *et al.*^48^ derived the depletion thickness around a sphere for a flexible chain (*l*_K_ = 1) using the full expression given in eqn (26). They obtained an equation that has nearly the same limits as eqn (32), the difference is in the numerical pre-factor of the third term, which is a factor π/3 smaller. We incorporate this limit and obtain the following expression for the depletion thickness around a sphere:33
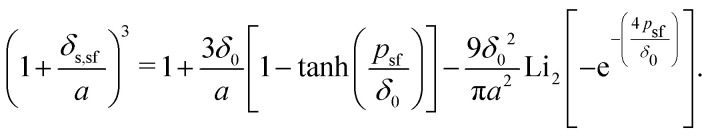


While all the equations in this section were written in terms of *δ*_0_, this can easily be extended towards the semidilute regime if the generalized depletion thickness is used as shown in the previous section. In that case, *δ*_0_ is replaced by 
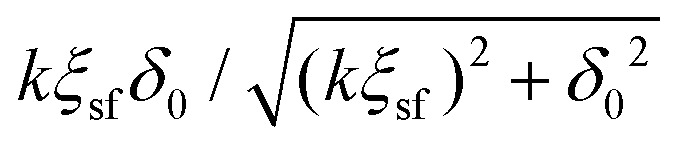
.

## 4 Results and discussion

### 4.1 Concentration profile near a flat wall

**Fig. 2:**
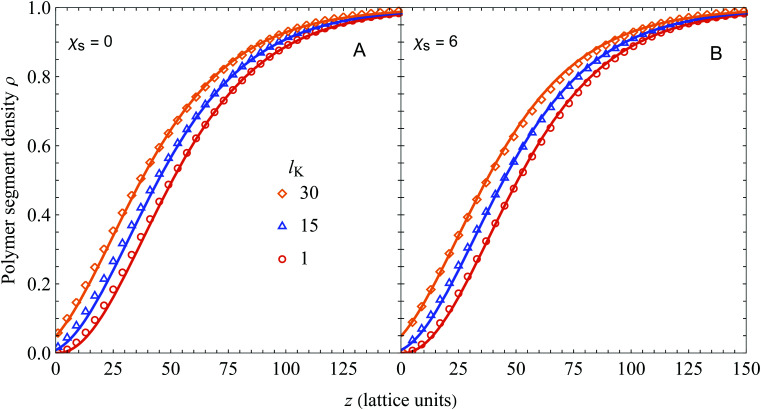
Polymer segment density profile near a hard wall for polymer solutions containing semi-flexible polymer chains, with *φ*_b_ = 10^−6^, *R*_g_ = 50, and *χ*_s_ = 0 (A) or *χ*_s_ = 6 (B). Our result (eqn (18), solid curves) is compared with numerical SCF calculations for a Kuhn length of 1 (circles), 15 (triangles), and 30 (diamonds), corresponding to a contour length (in units of the bond length) of 15 000, 1000, and 500, respectively.

In Fig. 2 we compare the analytical expression (solid curves, eqn (18)) with numerical SCF calculations (symbols). The segment density profiles *ρ*(*z*) = *φ*(*z*)/*φ*_b_ are plotted for different Kuhn lengths as indicated, and two different values of the adsorption energy *χ*_s_. The polymer bulk concentration *φ*_b_ was set at 10^−6^ (very dilute) and the radius of gyration *R*_g_ (in units of the monomer length) was fixed at 50 by varying the number of monomers *N*, using *N* = 6*R*_g_^2^/(*l*_K_) (thus varying the contour length). As can be seen, there is good agreement between eqn (18) and SCF results for a wide range of chain stiffnesses.

In the derivation of the continuum expression for semi-flexible chains the adsorption energy *χ*_s_ was neglected, while it is taken into account in the SCF computations. As follows from comparing Fig. 2A (*χ*_s_ = 0) and B (*χ*_s_ = 6), this assumption is accurate as *χ*_s_ hardly affects the density profiles. This observation was extensively discussed by Fleer and Skvortsov^38^ for flexible polymers; for *χ*_s_ much larger than the critical adsorption energy *χ*_s,crit_ there is barely any effect of *χ*_s_ on the concentration profile of depleted polymers. Closer to the critical adsorption energy there is a shift towards the surface with decreasing *χ*_s_. In principle it is possible to take this into account by defining another density shift length which depends solely on the adsorption energy, however in this work we focus on strong depletion. This observation also indicates the limitations of our presented theory: it is only valid sufficiently far away from the critical adsorption energy *χ*_s,crit_, which was derived by Birshtein *et al.*^21^ as 
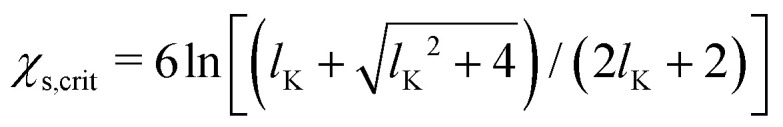
.

**Fig. 3:**
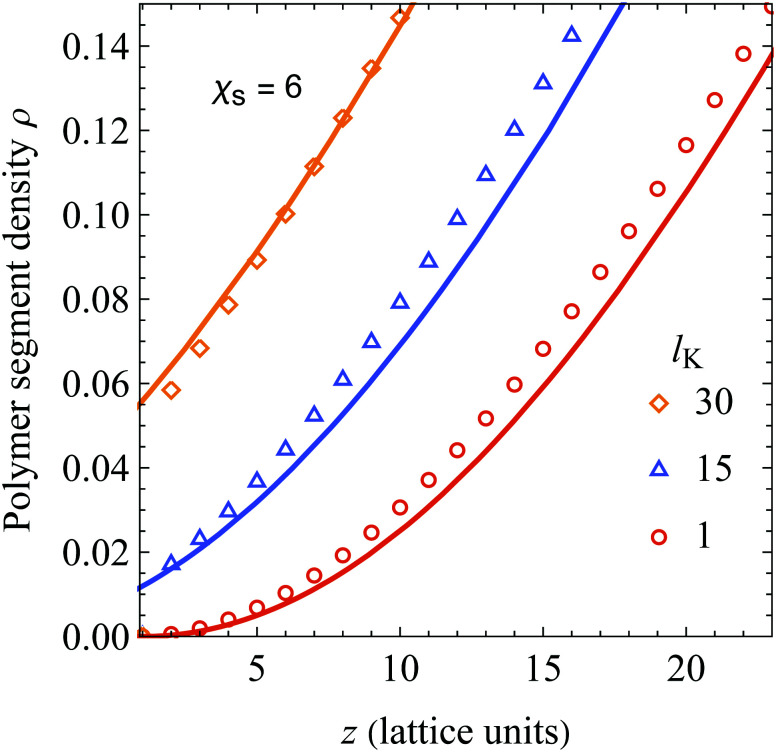
A close-up of Fig. 2 A around the layers close to the non-adsorbing surface. Our analytical result (eqn (18), solid curves) is compared with numerical SCF computations for a Kuhn length of 1 (circles), 15 (triangles), and 30 (diamonds).

Lastly, since *p*_sf_ > 0 for all *l*_K_ > 1, eqn (18) predicts a relatively large segment density at the surface. Fig. 3 shows details of the layers close to the surface from Fig. 2A. As can be seen, the agreement is good for all layers, except for the first. The deviation in the first layer can be explained by realizing that the adsorption energy is neglected in deriving of the density shift length. The deviations for layer *z* > 1 are small because *χ*_s_ is only felt by polymer segments in the first layer. Thus, the assumption of *u* = 0 hardly affects the density profiles for *z* > 1.

### 4.2 Depletion thickness at a flat wall

**Fig. 4:**
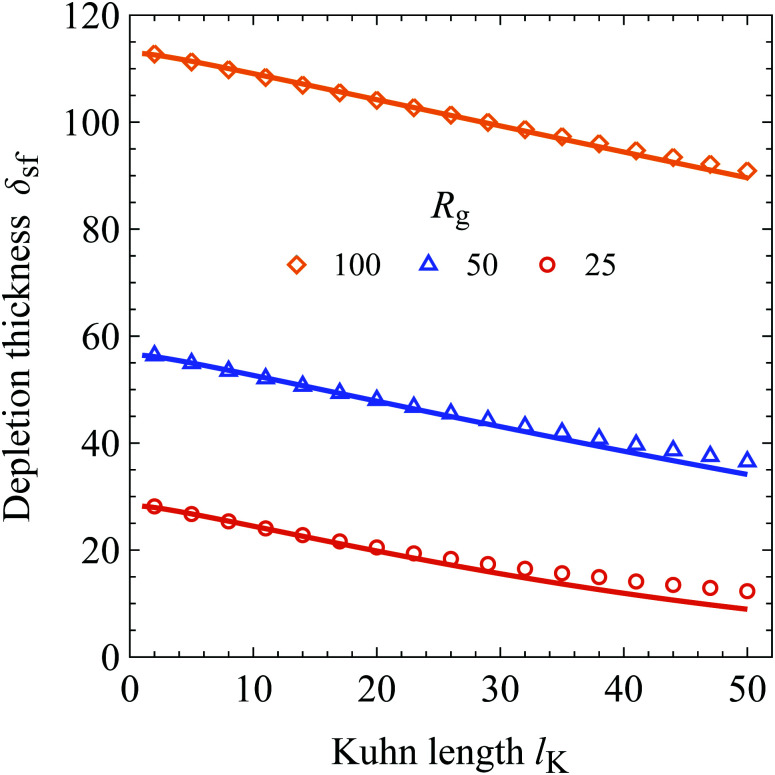
Chain stiffness dependence of the depletion thickness: *δ*_sf_ is plotted as a function of the Kuhn length *l*_K_ for three radii of gyration as indicated. Parameters: *χ*_s_ = 6 and *φ*_b_ = 10^−6^. The solid curves follow eqn (20).

In Fig. 4 the result of eqn (20) (solid curves) is compared to SCF results (symbols) as a function of the Kuhn length for the three values of *R*_g_ indicated in the plot. In order to keep *R*_g_ constant, the contour length (number of monomers *N*) is varied and rounded to the nearest integer. A bulk polymer segment volume fraction of *φ*_b_ = 10^−6^ and an adsorption energy of *χ*_s_ = 6 was used in the SCF calculations. As is shown, there is good agreement between the analytical expression for the depletion thickness at a wall in contact with solutions containing semi-flexible chains and the results obtained from SCF. Again, as expected from the concentration profile, the depletion thickness of a semi-flexible chain is smaller as compared to a flexible chain with the same radius of gyration. In turn, the depletion thickness becomes a monotonically decreasing function of chain stiffness if the radius of gyration is kept constant.

For the calculations where the polymers have a radius of gyration *R*_g_ of 25 and 50, the deviations between the predictions of eqn (20) and SCF results are small but slightly increase with increasing Kuhn length. These deviations can be explained because the number of Kuhn segments *N*_K_ becomes increasingly smaller for larger Kuhn lengths. As an example, in the case of *R*_g_ = 25 at *l*_K_ = 50, the number of Kuhn segments is *N*_K_ = 1.5. For a small number of Kuhn segments the physics of the polymer chain deviates from a statistical Gaussian chain and becomes more rigid-rod-like, which is not accounted for in continuum theories of polymer solutions. For *N*_K_ > 8 the error between the numerical SCF and the analytical theory is smaller than 5%.

### 4.3 Concentration dependence of the depletion thickness

**Fig. 5:**
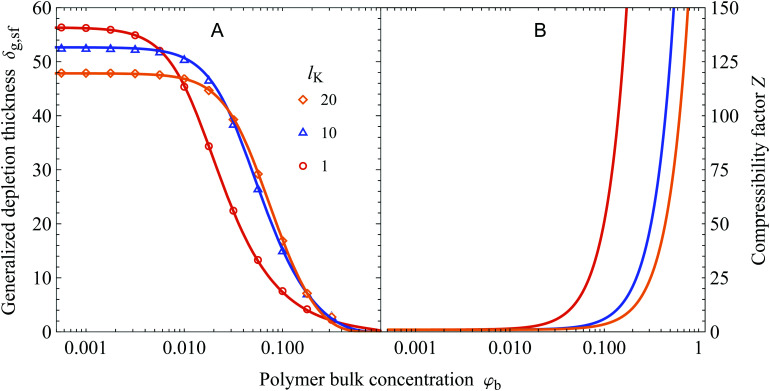
(A) The generalized depletion thickness as a function of the polymer bulk concentration for a solution of polymers with a radius of gyration of 50, contour lengths of the polymers (in units of the bond-length) are 15 000, 1500, and 750 and overlap concentrations of the polymers are *φ*_ov_ = 0.0286, *φ*_ov_ = 0.0029, and *φ*_ov_ = 0.0014 for *l*_K_ = 1, 10, and 20, respectively. The symbols are numerical SCF data and the solid curves are calculated using eqn (25). (B) The compressibility factor as defined in eqn (35) as a function of the polymer bulk concentration for polymers with a gyration radius of 50.

In Fig. 5A we show the concentration dependence of the depletion thickness of semi-flexible polymer solutions. The generalized depletion thickness is plotted as a function of the polymer bulk concentration for various Kuhn lengths, again for *R*_g_ = 50. The symbols are results from numerical SCF computations, and the solid curves are the predictions of eqn (25). An adsorption energy of *χ*_s_ = 6 is used in the SCF calculations. The polymer bulk concentration can be converted to concentrations in terms of fractions of the overlap concentration *ϕ*_b_ using *φ*_b_/*φ*_ov_ = *ϕ*_b_, where *φ*_ov_ = *N*/(4/3π*R*_g_^3^). For Kuhn lengths *l*_K_ = 1, *l*_K_ = 10, and *l*_K_ = 20 used in the Figure this corresponds to *φ*_ov_ = 0.0286, *φ*_ov_ = 0.0029, and *φ*_ov_ = 0.0014.

The general observation is that increasing the polymer segment bulk concentration above a certain concentration compresses the depletion zone. This can be understood by the increase in osmotic pressure with increasing polymer segment bulk volume fraction, which pushes polymer chains towards the non-adsorbing surface.^17,18^ In turn, the depletion thickness decreases. Eqn (25) is in good agreement with SCF calculations. A notable result is that increasing the chain stiffness results in a more extended concentration range where the depletion thickness is constant; the decrease of *δ*_g,sf_ shifted to higher polymer concentrations for solutions with stiffer polymer chains. Additionally, because the semi-flexible polymers have less configurational entropy, the depletion thickness is smaller in the dilute limit compared to the flexible case. These two effects result in a cross-over concentration, after which the solution containing more flexible polymers has a smaller depletion thickness than a solution with stiffer polymers.

The larger range of ‘dilute’ behavior can be explained by looking at the osmotic pressure *Π* of the polymer solution, which for polymer solutions in a *θ*-solvent is given by the approximate (mean-field) Flory–Huggins expression:^49,50^34
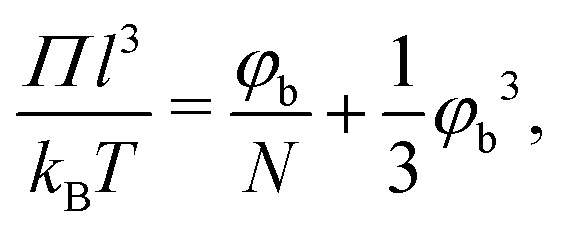
where *N* is the number of monomers in the polymer chain. In the dilute limit the osmotic pressure approaches the van 't Hoff limit *Π*_0_*l*^3^/*k*_B_*T* = *φ*_b_/*N*. Let us now define a compressibility factor *Z*:35
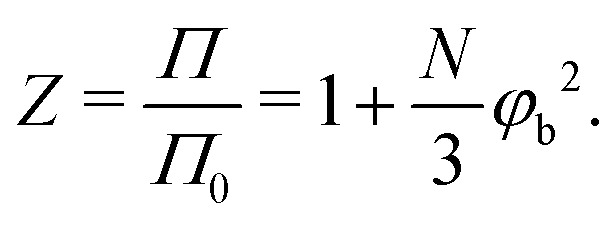
For polymers with a constant radius of gyration, increasing the Kuhn length scales down the number of monomers as *N* ∼ *R*_g_^2^/*l*_K_*l*^2^. Therefore, *Z* increases more for solutions containing flexible polymers than for solutions containing stiffer polymers. This phenomenon results in a lower polymer concentration at which *Z* increases sufficiently to facilitate the compression of the depletion layer, which is shown in Fig. 5B.

**Fig. 6:**
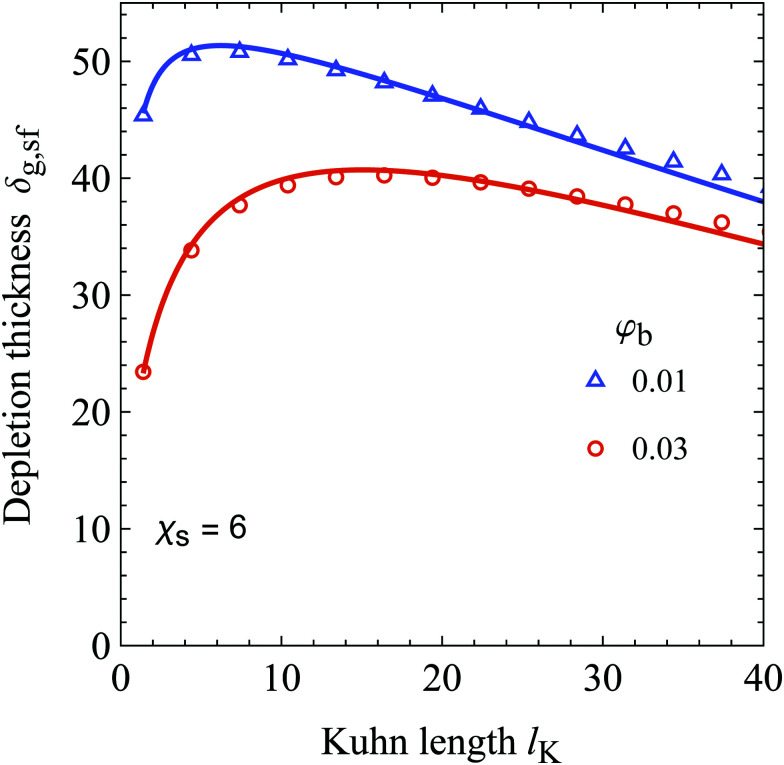
The generalized depletion thickness as a function of the Kuhn length for *φ*_b_ = 0.03 and *φ*_b_ = 0.01. The radius of gyration of the polymers is kept constant at 50. The symbols are numerical SCF data and the solid curves are our analytical expression, eqn (25).

The cross-over discussed earlier indicates the existence of a Kuhn length at which the depletion thickness has a maximum at fixed polymer bulk concentration; this is shown in Fig. 6. Here the generalized depletion thickness in the semidilute regime is plotted as a function of the Kuhn length for a bulk concentration of *φ*_b_ = 0.03 and *φ*_b_ = 0.01. We now indeed find a maximum as a function of both the polymer bulk concentration *φ*_b_ and the Kuhn length *l*_K_. The previously mentioned effects can explain this maximum; a solution containing stiffer polymers behaves quasi-ideal up to higher concentrations because the coils are more dilute. On the other hand, because of the decrease in configurational entropy, the depletion thickness in the dilute limit is smaller for solutions containing more stiff polymers. The balance between these two effects results in a maximum of the depletion thickness. This maximum also has important implications for the interactions between colloids in colloid–polymer mixtures. The concentration dependent decrease of the depletion thickness in the semidilute regime has a major impact on the location of the triple point, as shown by Fleer *et al.*^51^ As we observed a maximum in the depletion thickness as a function of chain stiffness, one might expect that chain stiffness thus also impacts the location of the triple point. However, the specific implications of chain stiffness on the phase behavior of polymer–colloid mixtures is out of scope of this work.

It is known that concentrated semi-flexible polymer solutions undergo an isotropic–nematic phase transition at sufficiently high chain stiffness.^45,52–54^ This effect is neglected in our theory, which limits our work to polymer concentrations below the isotropic-nematic phase transition. Semenov and Khokhlov showed^55,56^ that in the limit of *N* ≫ *l*_K_ a semi-flexible polymer solution is isotropic for *φ*_b_ ≲ 10.48/*l*_K_. In the case displayed in Fig. 5, this corresponds to *φ*_b_ ≲ 0.5 for *l*_K_ = 20 and *φ*_b_ ≲ 1 for *l*_K_ = 10. However, semi-flexible polymers near a non-adsorbing surface also show nematic ordering close to the surface.^57–60^ While this effect is also present for flexible polymers, it is much more pronounced for semi-flexible polymers with some form of intrinsic stiffness.^58,60^ This confinement-based nematic ordering results in an ordered layer with a thickness on the order of the persistence length of the polymer.^57–60^ Zhang *et al.* showed^60^ that within a lattice-based self-consistent field model for semi-flexible polymers, neglecting the free energy gain of nematization through a nematic aligning potential results in an underestimation of the effective ordering parameter of the polymers. Incorporating the nematic aligning potential would thus result in a further decrease of the depletion thickness, as the polymer segment density is increased near the surface due to the ordering of the chains. While it is possible to take bond-correlations into account within the Scheutjens-Fleer SCF formalism,^32,39^ it does not lead to tractable analytical equations, thus we did not include this in our theory. Nevertheless, we expect that there is qualitative agreement with models that do include the nematic field coupling of the polymer chains. Additionally, we must note that the nematic ordering of semi-flexible polymers is significantly enhanced when the polymers are confined between two surfaces, thus careful consideration of the approximate character of the presented theory must be taken into account when describing, for example, depletion interactions between two surfaces.

### 4.4 Depletion thickness around a sphere

**Fig. 7:**
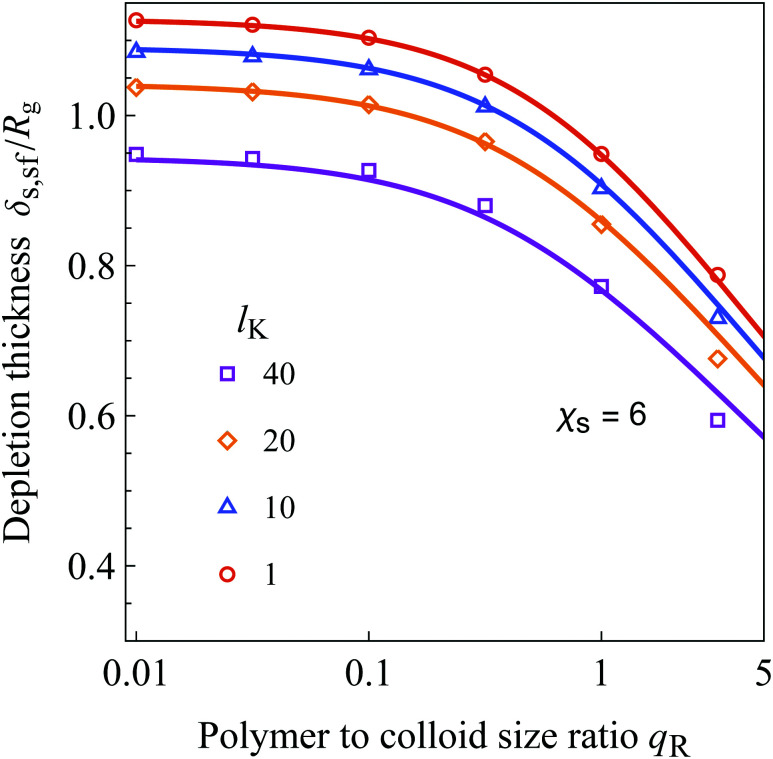
The depletion thickness around a sphere as a function of the polymer to colloid size ratio *q*_R_ for *R*_g_ = 100 and a variable colloidal radius *a*. Results are shown for *l*_K_ = 1, 10, 20 and 40, corresponding to a contour length (in units of the bond-length) of 60 000, 6000, 3000, and 1500, respectively. The solid curves are predictions of eqn (33) and the symbols correspond to results from SCF computations using a polymer bulk concentration of *φ*_b_ = 10^−6^.

Fig. 7 shows the depletion thickness around a sphere (eqn (33)) as a function of the polymer–colloid size ratio *q*_R_ = *R*_g_/*a*. We compare the analytical equation (solid curves) with numerical SCF calculations (symbols). The radius of gyration of the polymers (in units of the monomer length) is *R*_g_ = 100, the Kuhn length is varied from 1 to 40, and the effects of colloidal sphere radius are scanned from *a* = 10 000 to *a* = 10, where *a* is again in units of the bond length *l*. To obtain the numerical SCF data a polymer bulk concentration of *φ*_b_ = 10^−6^ and *χ*_s_ = 6 was used.

Also, for spherical geometry, the depletion thickness decreases for solutions containing stiffer chains. In the limit of *q*_R_ → 0 the exact flat plate result is re-obtained as expected. When *q*_R_ increases, the depletion thickness decreases as a result of an increase in the number of polymer configurations near the curved surface. When the polymers are large compared to the spheres, configurations where the polymer is wrapped around the colloidal sphere are possible.^16^ This increase in the number of configurations results in a smaller perturbation of the polymer solutions and thus a smaller depletion thickness. Intuitively, chain stiffness also has an effect on the number of available configurations of the polymer. Remarkably, it is observed that incorporating the density shift length *p*_sf_ accounts accurately for this effect on the number of configurations, even in spherical geometry.

It can be observed that eqn (33) starts to deviate considerably from the SCF data for *q*_R_ > 2. This is due to the less accurate approach of using eqn (29) for large *q*_R_, as noted by Fleer *et al.*^18^ and Tuinier and Lekkerkerker.^61^ While corrections to the concentration profile, and therefore the depletion thickness, are possible,^61^ they do not result in analytical equations for the depletion thickness; thus, we are satisfied with the current approximate result. We note that for polymer solutions in *θ*-solvents in the semidilute regime, eqn (33) overestimates the depletion thickness around a sphere up to 10% at large values of *q*_R_. This is because the tanh(*z*/*δ*_0_) approximation in eqn (29) is strictly speaking not accurate for *θ*-solvent conditions, as noted by Fleer *et al.*^18^ This can be corrected by replacing tanh(*z*/*δ*_0_) with a more involved function, accounting more accurately for the energetic interactions between the solvent and polymer segments.

## 5 Conclusions

In this paper simple, yet accurate analytical expressions for the concentration profile and depletion thickness of solutions containing semi-flexible polymers near a hard flat plate and near a sphere have been derived. Using previous results for solutions of flexible polymers and surface boundary conditions derived from self-consistent field theory, we introduced a density shift length *p*_sf_ which accounts for the decrease in configurational entropy of a semi-flexible polymer. The resulting theoretical predictions quantitatively describe the segment density profiles from self-consistent field lattice computations for solutions containing semi-flexible polymer chains. The general finding was that stiffer chains lead to a decrease of the width of the depletion zone.

Using the standard definition of the depletion thickness we derived that the depletion thickness *δ*_sf_ of a solution containing semi-flexible polymers has a simple form; *δ*_sf_ = *δ*_0_[1 − tanh(*p*_sf_/*δ*_0_)], with 
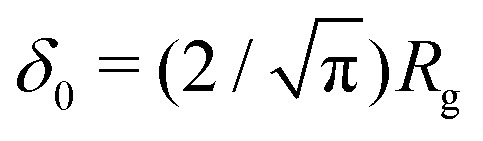
 and *R*_g_ the radius of gyration of the polymers. This approach was extended towards spherical geometry with similar findings: stiffer chains in solution lead to smaller depletion zones. Also, in this case, the theoretical predictions describe the numerical SCF calculations very well, except for the case where the polymers are much larger than the spheres.

Above a certain polymer concentration the depletion thickness drops with increasing polymer concentration. We found that solutions containing stiffer polymers behave quasi-ideal (*δ* ≈ *δ*_0_) over a more extended range of concentrations. Furthermore, it was shown that there is a local maximum in the depletion thickness as a function of chain stiffness in the semidilute regime. This maximum may have important implications for the phase behavior of colloid–polymer mixtures.

The mean-field treatment presented in this article is built upon the mean-field theory for flexible polymers. The latter has its limitations; both within the self-consistent field calculations and the analytical expressions fluctuations are not accounted for. This means that the scaling exponents for the correlation length differ from more accurate treatments such as scaling theory or renormalization group theory (RGT) calculations. However, the results shown here provide significant insight in the effect of chain stiffness on the depletion behavior of polymer solutions. As a next step, for good-solvent conditions, chain swelling, and fluctuations in the semidilute regime should be incorporated in the presented theory. One possible methodology is by using the correct scaling exponents obtained from scaling theory or RGT calculations.^51^

One of the main benefits of the new theory is that concentration effects in the semidilute regime are taken into account. Furthermore, it is entirely consistent with the theory of depletion for solutions of flexible polymers, enabling a general theoretical approach that extends towards stiffer polymer chains. A useful application of this new theory is to predict the phase behavior of mixtures of semi-flexible polymers and colloids, which we intend to work on in the future.

## Conflicts of interest

There are no conflicts to declare.

## Supplementary Material

## Figures and Tables

**Fig. 8:**
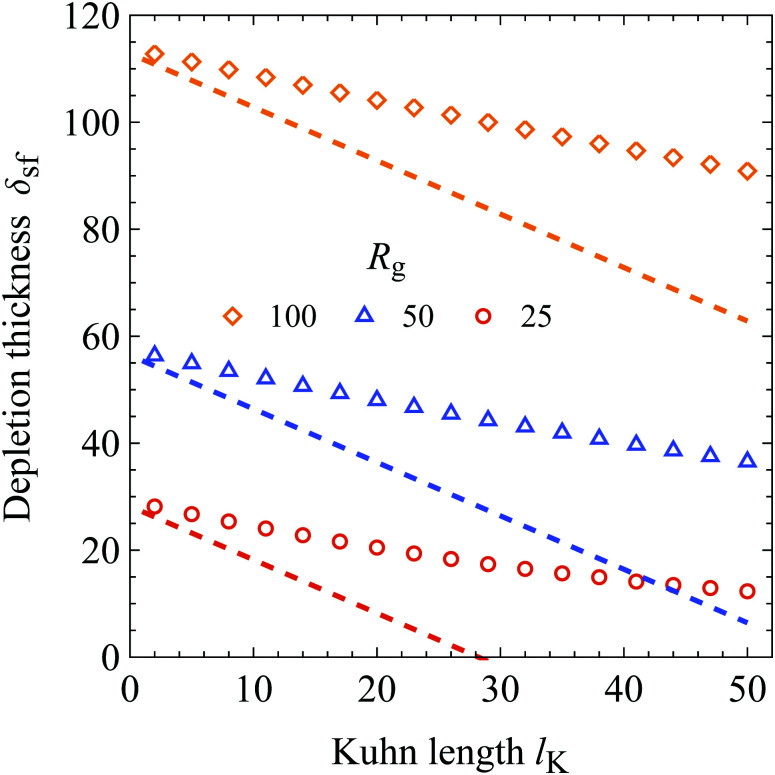
Chain stiffness dependence of the depletion thickness. *δ*_sf_ is plotted as a function of the Kuhn length *l*_K_ for three radii of gyration as indicated, *χ*_s_ = 6 and *φ*_b_ = 10^−6^. The dashed curves are the result of Ausseré *et al.*,^29^*δ*_sf_ = *δ*_0_ − *l*_K_, and the symbols are numerical SCF computations.

**Fig. 9:**
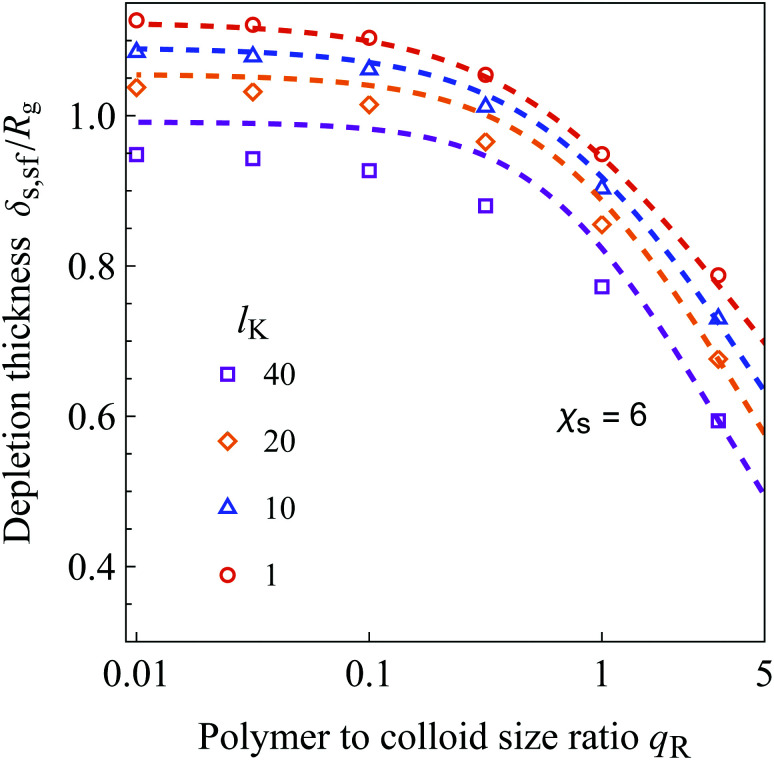
The depletion thickness around a sphere as a function of the polymer to colloid size ratio *q*_R_ for *R*_g_ = 100 and a variable colloidal radius *a*. A Kuhn length of 1, 10, 20 and 40 is shown, corresponding to a contour length (in units of the bond-length) of 60 000, 6000, 3000, and 1500, respectively. The dashed curves are the Padé approximation of Lue^30^ converted to a depletion thickness by Tuinier,^62^ and the symbols are SCF calculations. A polymer bulk concentration of *φ*_b_ = 10^−6^ was used for the SCF calculations.

**Fig. 10:**
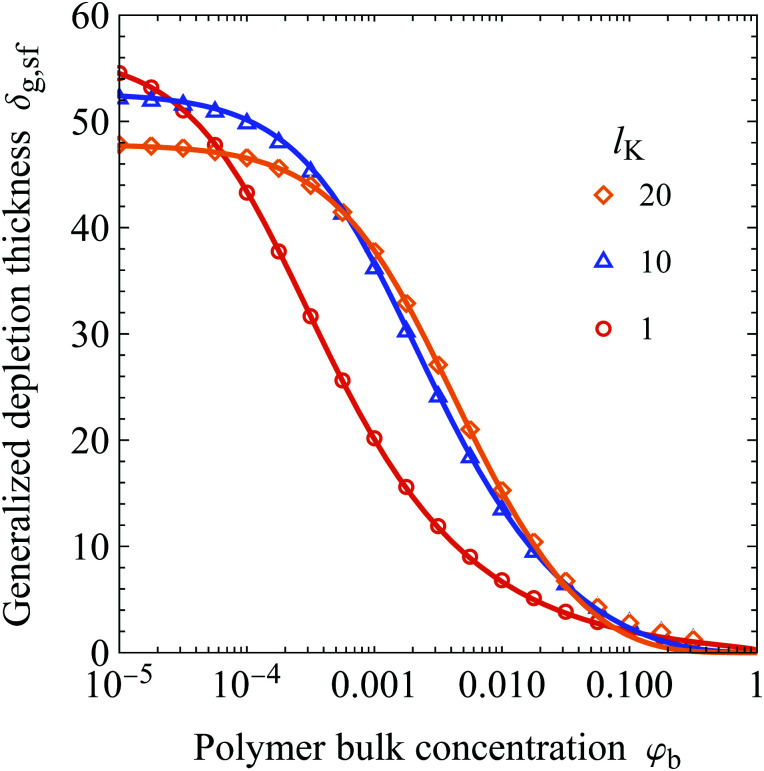
The generalized depletion thickness in a good-solvent as a function of the polymer bulk concentration for polymers with *R*_g_ = 50, contour lengths of the polymers (in units of the bond-length) are 15 000, 1500, and 750 for *l*_K_ = 1, 10, and 20, respectively. The symbols are numerical SCF data and the solid curves are calculated using eqn (25) with *χ* = 0.

## Appendices

### A Comparison with earlier theories

In the introduction of this paper we noted that the theories of Ausseré *et al.*^29^ and Lue^30^ are inconsistent with numerical SCF calculations. In this appendix we reproduce Fig. 4 and 7 with dashed lines that represent the expressions obtained by Ausseré *et al.*^29^ and Lue,^30^ respectively. It must be noted that Lue did not derive an expression for depletion thickness but rather for the excluded volume; Tuinier used this to obtain an expression for the depletion thickness,^62^ which is what is shown.

The first comparison is shown in Fig. 8; here the depletion thickness is plotted as a function of the Kuhn length for three radii of gyration as indicated. The dashed lines are the expression of Ausseré *et al.*,^29^*δ*_sf_ = *δ*_0_ − *l*_K_, and the symbols are numerical SCF calculations. The SCF calculations are obtained using *φ*_b_ = 10^−6^ and *χ*_s_ = 6. As can be seen, the analytical approximation starts to deviate from the SCF calculations substantially at larger Kuhn lengths.

Next, we compare the results of Lue^30^ to SCF computations in Fig. 9. Here we plot the depletion thickness around a sphere as a function of the polymer to colloid size ratio *q*_R_. The dashed curves again are the analytical approximation, and the symbols are numerical SCF calculations. Remarkably, for smaller *q*_R_ the analytical approximation overestimates the depletion thickness for all *l*_K_ > 1, while for large *q*_R_ it is consistent with the SCF calculations.

### B Analytical approximation for the spherical depletion thickness

In order to obtain an analytical equation which can be used in further theoretical investigations, the excluded volume integral, eqn (31), must be solved approximately. This approximation can be done as follows. We can change the equations for the concentration profile such that it closely approximates eqn (30) but has a closed-form solution to the excluded volume integral. If we then match the flat plate limit and flexible chain limit it approximates the numerical integral fairly well. Through trail-and-error it was found that one such function is:

36

This function can be used as an input in the excluded volume integral and has the solution:

37

We find that in the limit of *a* → ∞ this equation results in *δ*_sf_ = *δ*_0_[1 − tanh(2*p*_sf_/*δ*_0_)]. To match the flat plate result in this limit we have to replace 2*p*_sf_ → *p*_sf_ in the second term of eqn (37) and thus we end up with eqn (32).

### C The depletion thickness in a good solvent

In this section we check the validity of eqn (25) for the good-solvent case (*χ* < 0.5). It must be noted that within the mean-field treatment presented in this article, chain swelling and the *χ*-dependency of the concentration-independent depletion thickness *δ*_0_ is not taken into account.^18^ Hanke *et al.* found^36^ that for excluded volume polymers *δ*_0_ = 1.07*R*_g_, where *R*_g_ scales with *N*_k_^0.588^.

In Fig. 10 the result of eqn (25) (solid curves) is compared to SCF results (symbols) as a function of *φ*_b_ for three values of *l*_k_ as indicated in the plot. The polymer radius of gyration is 50 and the solvency parameter *χ* = 0, thus we are in the good-solvent limit of pure excluded volume interactions. As can be seen, the agreement is quantitative within the mean-field approximation used. We find similar trends as discussed in Section 4.3: with increasing polymer segment bulk concentration the depletion zone is compressed. This effect is more pronounced in good-solvent conditions as compared to the *θ*-solvent condition, as was first shown by Fleer *et al.*^18^ Interestingly enough the cross-over concentrations are recovered, thus, there is also a maximum in the depletion thickness in good-solvent conditions. This leads us to the conclusion that within a mean-field approximation, the existence of the maximum is a semi-flexible polymer specific effect, independent of the solvency.
